# Effects of dobutamine and phenylephrine on cerebral perfusion in patients undergoing cerebral bypass surgery: a randomised crossover trial

**DOI:** 10.1016/j.bja.2020.05.040

**Published:** 2020-07-24

**Authors:** Annemarie Akkermans, Judith A.R. van Waes, Tristan P.C. van Doormaal, Eric E.C. de Waal, Gabriël J.E. Rinkel, Albert van der Zwan, Cor J. Kalkman, Wilton A. van Klei

**Affiliations:** 1 Department of Anaesthesiology, University Medical Centre Utrecht, Utrecht University, Utrecht, the Netherlands; 2 Department of Neurology and Neurosurgery, Brain Centre, University Medical Centre Utrecht, Utrecht University, Utrecht, the Netherlands; 3 Department of Neurosurgery, University Hospital Zürich, Zurich, Switzerland

**Keywords:** blood pressure, cardiac output, cerebral bypass surgery, cerebral ischaemia, cerebral perfusion

## Abstract

**Background:**

Patients undergoing cerebral bypass surgery are prone to cerebral hypoperfusion. Currently, arterial blood pressure is often increased with vasopressors to prevent cerebral ischaemia. However, this might cause vasoconstriction of the graft and cerebral vasculature and decrease perfusion. We hypothesised that cardiac output, rather than arterial blood pressure, is essential for adequate perfusion and aimed to determine whether dobutamine administration resulted in greater graft perfusion than phenylephrine administration.

**Methods:**

This randomised crossover study included 10 adult patients undergoing cerebral bypass surgery. Intraoperatively, patients randomly and sequentially received dobutamine to increase cardiac index or phenylephrine to increase mean arterial pressure (MAP). An increase of >10% in cardiac index or >10% in MAP was targeted, respectively. Before both interventions, a reference phase was implemented. The primary outcome was the absolute difference in graft flow between the reference and intervention phase. We compared the absolute flow difference between each intervention and constructed a random-effect linear regression model to explore treatment and carry-over effects.

**Results:**

Graft flow increased with a median of 4.1 (inter-quartile range [IQR], 1.7–12.0] ml min^−1^) after dobutamine administration and 3.6 [IQR, 1.3–7.8] ml min^−1^ after phenylephrine administration (difference –0.6 ml min^−1^; 95% confidence interval [CI], –14.5 to 5.3; *P*=0.441). There was no treatment effect (0.9 ml min^−1^; 95% CI, 0.0–20.1; *P*=0.944) and no carry-over effect.

**Conclusions:**

Both dobutamine and phenylephrine increased graft flow during cerebral bypass surgery, without a preference for one method over the other.

**Clinical trial registration:**

Netherlands Trial Register, NL7077 (https://www.trialregister.nl/trial/7077).

**Editor's key points**

• There is debate whether an increase in blood pressure or cardiac output is more effective in cerebral blood flow augmentation during neurosurgery.
• In this study of cerebral bypass surgery, it was hypothesised that an increase in cardiac output would lead to higher graft perfusion than an increase in blood pressure.
• Cardiac output was increased by dobutamine and blood pressure by phenylephrine following a randomised crossover design.
• Either drug increased cerebral graft flow to a similar degree.
• This small study warrants further evaluation.

Preservation of adequate cerebral perfusion during cerebral bypass procedures is a challenge for both neurosurgeons and anaesthesiologists.^1^ Cerebral bypass surgery can be used as a revascularisation technique for flow augmentation in steno-occlusive vascular disease such as moyamoya disease, or flow preservation when a major artery has to be sacrificed to treat an underlying disease such as a complex intracranial aneurysm.^1^ Graft patency rates are generally well above 90%.^2^ However, graft patency itself does not guarantee adequate cerebral perfusion, and conventional cerebral bypass surgery carries a risk of intraoperative ischaemic stroke.^1^ Therefore, it has been suggested to maintain a normal blood pressure during general anaesthesia or to even increase the blood pressure with 10–20% from preoperative baseline.^3^ To achieve this goal, the administration of vasopressors is often required.^3^ Interestingly, systolic blood pressure (SBP) levels were not associated with graft flow in the postoperative setting.^4^ Concurrent administration of vasopressors might partly explain this observation, as vasoconstriction can actually decrease blood flow. An increase in blood pressure with vasopressors might surpass the effect of vasoconstriction and eventually increase the cerebral perfusion, but at the cost of systemic hypertension. However, as systemic blood pressure is determined by cardiac output and total peripheral resistance, it can be argued that an increase in graft flow can also be accomplished by an increase in cardiac output with the use of inotropes, without the side-effects of vasoconstriction and systemic hypertension.^5^ Although according to Ohm's law, augmenting cardiac output should not increase cerebral blood flow when the blood pressure remains unchanged, this axiom assumes that we know the pressure at the level of small cerebral arteries. However, in a hypovolaemic patient, vasopressors can increase central blood pressure to normal levels while at the same time there is considerably impaired organ perfusion.^6^^,^^7^ Unfortunately, the differential effect of blood pressure or cardiac output augmentation on cerebral blood flow during neurosurgery has hardly been studied.^8^, ^9^, ^10^, ^11^, ^12^

We hypothesised that inotropes (to increase cardiac output) rather than vasopressors (to increase blood pressure) are a key element for adequate graft flow and cerebral perfusion. Thus, we aimed to study the effect dobutamine administration *vs* the effect of phenylephrine administration on graft perfusion in patients undergoing cerebral bypass surgery.

## Methods

### Study design

This randomised crossover study was conducted between September 2018 and July 2019 at the University Medical Centre (UMC) Utrecht in adherence to the Consolidated Standards of Reporting Trials (CONSORT) statement: extension to randomised crossover trials.^13^ The local medical ethics committee, the national competent authority and the European Medicines Agency approved the study protocol (UMC Utrecht Medical Research Ethics Committee 18/321, Protocol number NL65095.041.18 and EudraCT number 2018-002008-15). This trial was registered at the Netherlands Trial Register (NL7077; Principal Investigator: W.A. van Klei; registration date: June 21, 2018). The full study protocol is available upon request.

**Fig 1:**
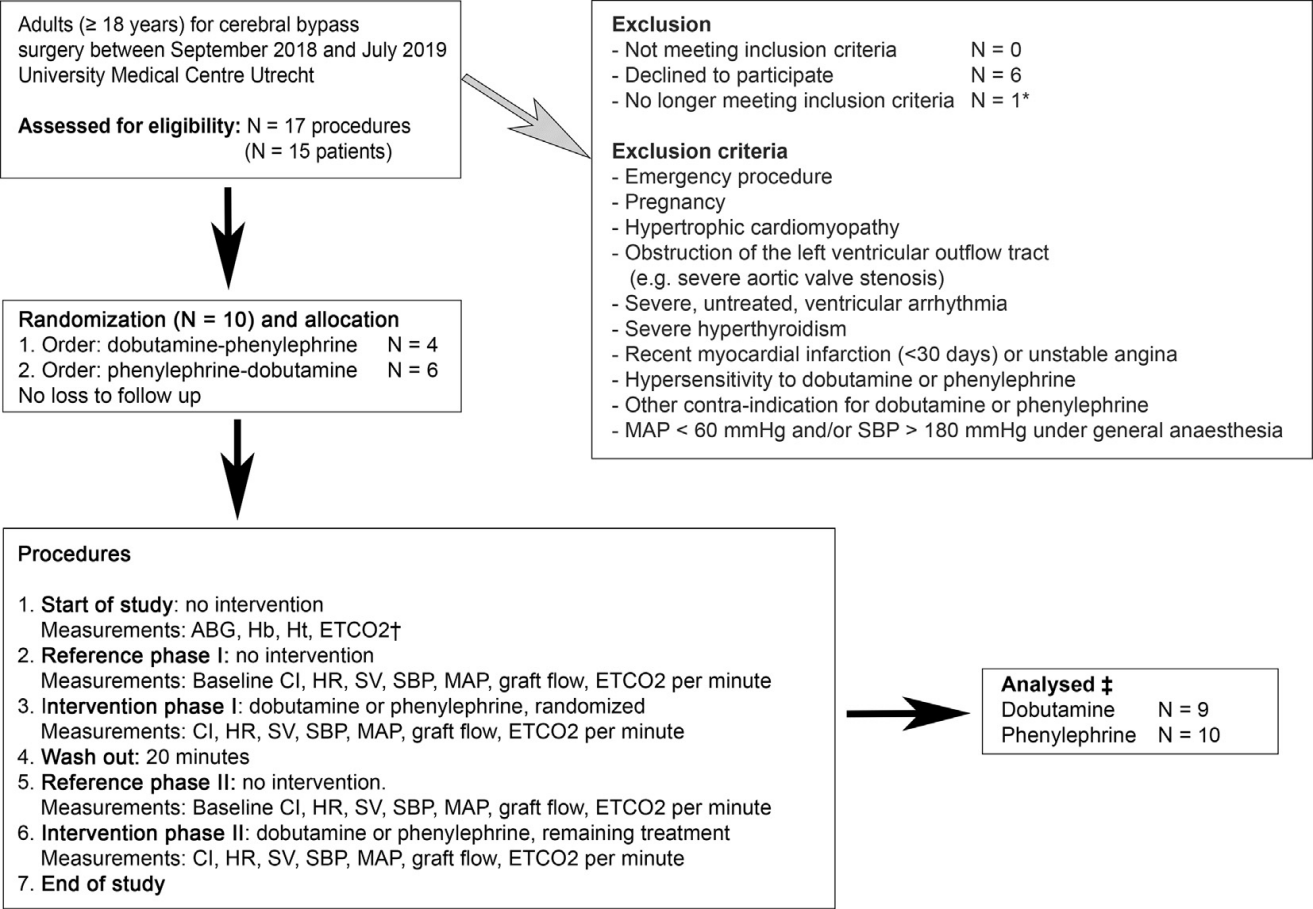
Study design and flowchart. ∗Drop-out before randomisation: one patient signed informed consent, but during the procedure primary clipping of the giant cerebral aneurysm was possible and an intracranial–intracranial cerebral bypass was no longer necessary. ^†^ETCO_2_ value that corresponds to *P*aco_2_ (arterial carbon dioxide pressure) from arterial blood gas sampling. ^‡^One patient developed arrhythmia after dobutamine administration. We did not exclude this patient entirely, but only excluded data obtained during the dobutamine intervention from our analyses. ABG, arterial blood gas; CI, cardiac index; ETCO_2_, end-tidal carbon dioxide; Hb, haemoglobin; HR, heart rate; Ht, haematocrit; MAP, mean arterial pressure; SBP, systolic blood pressure; SV, stroke volume.

Adult patients (≥18 yr) presenting for an extracranial–intracranial or intracranial–intracranial cerebral bypass were eligible for inclusion after written informed consent was obtained, irrespective of the indication or type of graft. Exclusion criteria were an emergency procedure, pregnancy, a contraindication for either dobutamine or phenylephrine, and MAP <60 mm Hg or SBP >180 mm Hg under general anaesthesia before the start of the interventions (Fig. 1). Patients could be included a second time when undergoing surgery on the contralateral side.

The interventions took place after construction of the bypass. Patients were randomised to sequentially receive dobutamine and phenylephrine via a central venous catheter (Fig. 1). Randomisation to determine which drug was to be given first was performed using sealed opaque envelopes in a 1:1 allocation ratio. The attending anaesthetist opened the envelope at the end of the cerebral bypass procedure. After a first reference phase to record baseline graft flow, the first intervention (administration of dobutamine or phenylephrine) was applied. After a wash-out period of 20 min and a second reference phase, the alternative intervention was applied (Fig. 1). The dosages of dobutamine (2–15 μg kg^−1^ min^−1^) and phenylephrine (0.15–1 μg kg^−1^ min^−1^) varied depending on their effect on cardiac index and blood pressure, respectively. For dobutamine, the infusion rate was targeted at an increase in cardiac index of at least 10%, as compared with the mean cardiac index in the reference phase. For phenylephrine, the infusion rate was adjusted to target a 10% increase in MAP as compared with the reference phase. During the reference and the intervention phases SBP, MAP, heart rate, stroke volume, cardiac index, and graft flow were measured every minute once a steady state was reached for at least 2 min.

Infusion of fluids and administration of other medications was kept constant throughout the study period. To maintain a constant arterial carbon dioxide pressure (*P*aco_2_), no adjustments to ventilator settings were allowed and end-tidal carbon dioxide (ETCO_2_) values were documented throughout the study period. To prevent vasoconstriction, topical application of papaverine at the intracranial part of the bypass was allowed throughout the measurement period and at the discretion of the neurosurgeon.

SBP, MAP, and heart rate were continuously measured via an arterial catheter using a fourth-generation FloTrac® transducer placed in the radial artery and an EV1000 monitor (Edwards Lifesciences, Irvine, CA, USA), and uncalibrated arterial pressure waveform analysis was used to measure stroke volume and cardiac index.^14^ Graft flow was measured with an ultrasonographic flow meter (Transonic Systems Inc., Ithaca, NY, USA), with a probe encircling the bypass in close proximity to the anastomosis with the intracranial artery.

The anaesthesiologist was blinded for graft flow, whereas the neurosurgeon, who measured graft flow, was blinded for medication given and blood pressure and cardiac index data.

### Conduct of general anaesthesia

General anaesthesia was maintained with propofol (induction bolus 1–3 mg kg^−1^, maintenance infusion 4–10 mg kg^−1^ h^−1^), remifentanil (0.25–0.5 μg kg^−1^ min^−1^), and atracurium (induction bolus 0.5 mg kg^−1^, maintenance infusion 5–10 μg kg^−1^ min^−1^). Management of blood pressure and ETCO_2_ outside the study period were left to the judgement of the attending anaesthesiologist, except that our local protocol prescribed to maintain SBP <180 mm Hg and to keep ETCO_2_ between 4.7 and 6.0 kPa. During anaesthesia but outside the study period, episodes of hypotension were treated with ephedrine (5 mg ml^−1^, bolus 2.5–10 mg) or phenylephrine (100 μg ml^−1^, bolus 100 μg, infusion 0.15–1 μg kg^−1^ min^−1^). When phenylephrine infusion was started before the start of the study, this was continued at a constant infusion rate during the study period. The intervention drug, either dobutamine or phenylephrine, was given in addition to this maintenance infusion. Only during the wash-out period, was a bolus or change in maintenance infusion of phenylephrine allowed and this was left to the judgement of the attending anaesthesiologist.

### Outcome measures

The primary outcome measure was the absolute change in graft flow during dobutamine and phenylephrine administration as compared with the reference phase. The secondary outcome measures were the change in MAP, SBP, heart rate, stroke volume, and cardiac index.

### Additional data collection

Data on patient, procedure, and graft characteristics were collected from electronic medical files. Variables were selected on the basis of their possible influence on graft patency or flow. Variables on patient and procedure characteristics included age, sex, BMI, ASA Physical Status Classification,^15^ indication and duration of the procedure, bypass technique, graft type (venous or arterial),^4^ donor vessel, and recipient vessel.^4^^,^^16^ In addition, data on comorbidities, use of cardiovascular medication, and intraoperative data were collected. Baseline MAP was defined as mean MAP from all values obtained within 5 min before induction, measured with an arterial catheter.

Before the first reference phase, an arterial blood gas sample was obtained and the corresponding ETCO_2_ level was documented to determine the gradient between *P*aco_2_ and ETCO_2_. In addition, haemoglobin and haematocrit were determined. This blood gas sampling was used to determine whether any deviations in *P*aco_2_, haemoglobin, or haematocrit were present that could possibly influence graft flow.

Finally, the amount of dobutamine and phenylephrine administered was collected, and the duration of the administration was noted.

### Sample size

As no data were available on the effect of an increase in cardiac index on graft perfusion in cerebral bypass surgery, we were not able to perform a proper sample size calculation. However, the crossover design enabled us to limit the sample size. With ∼20 cerebral bypass procedures in adults in our institution each year, we aimed to include 10 patients.

### Statistical analysis

All analyses were performed with use of R (Version 3.6.1– © [2019-07-05] for Macintosh, R, Inc., Vienna, Austria).^17^ Descriptive statistics were done using frequencies, percentages and either means with standard deviation (sd) or medians with 25th and 75th percentiles (inter-quartile range [IQR]) as appropriate.

The mean graft flow was estimated for each reference and intervention phase and was plotted over time for each patient. The change in graft flow between intervention phase I and the corresponding reference phase I, and between intervention phase II and reference phase II was calculated. Afterwards, a two-sided Wilcoxon signed rank test was used to assess the difference in flow, after confirmation that the data were not normally distributed. A pseudo-median was reported as differences between paired samples were not fully symmetrically distributed around the median. The same method was applied to assess differences in MAP, SBP, heart rate, stroke volume, and cardiac index.

To study the treatment effect (i.e. dobutamine *vs* phenylephrine), the sequence effect (i.e. randomisation order), and a potential carry-over effect (i.e. the duration of the wash-out period), a random-effect multivariable linear regression model was constructed, with graft flow as the dependent variable and treatment (dobutamine *vs* phenylephrine) as a fixed effect. Subject ID was included as a random effect to account for within-subject variance. As graft flow may increase between opening of the bypass and the end of surgery,^16^ we incorporated two reference phases in our study design. To account for any additional effect of timing of graft flow measurement, time was also included as random effect. We included an interaction term for treatment and randomisation order as a fixed effect to adjust for any incomplete wash-out.

*P*-values <0.05 were considered statistically significant and 95% confidence intervals (CI) were reported. There was no need to account for multiplicity.

## Results

Within the study period, 15 patients presented for cerebral bypass surgery for a total of 17 procedures. Of these, eight patients were enrolled and randomised to either receive first dobutamine and thereafter phenylephrine or *vice versa* (Fig. 1). Two patients were enrolled for a second time when they underwent surgery contralateral to the side of the first bypass. One patient developed a short episode of atrial arrhythmia when dobutamine was administered, which converted to sinus rhythm after discontinuation of dobutamine. Although there were no haemodynamic consequences, the validity of arterial wave form analysis might be compromised.^14^ Data obtained during dobutamine administration in this patient were removed from our analyses. There were no additional missing data. All patients received an extracranial–intracranial bypass, all with the superficial temporal artery as donor vessel and the middle cerebral artery as recipient vessel. Five patients (63%) were diagnosed with moyamoya disease of whom two were included twice, and three patients (38%) had atherosclerotic carotid artery occlusion. Additional baseline characteristics are presented in Table 1. There were no important deviations in *P*aco_2_, haemoglobin, or haematocrit.

**Table 1:** Baseline characteristics. ∗Only the first case per patient was included in this part of the table. ^†^This includes all 10 cases, including the two patients who presented twice for cerebral bypass surgery. ETCO_2_, end-tidal carbon dioxide; IQR, inter-quartile range.

Preoperative characteristics		Patients (*N*=8)∗	
Gender (%)	Female	5 (62.5)	
Age (yr, median [IQR])		48 [41–53]	
BMI (kg m^−1^, median [IQR])		29 [26–35]	
ASA physical status (%)	1	0 (0.0)	
	2	2 (25.0)	
	3	5 (62.5)	
	4	1 (12.5)	
Ischaemic heart disease (%)		0 (0.0)	
Heart failure (%)		0 (0.0)	
Cerebrovascular accident (%)	Ischaemic	8 (100.0)	
	Haemorrhagic	0 (0.0)	
Diabetes mellitus (%)	No	7 (87.5)	
	Non-insulin dependent	0 (0.0)	
	Insulin dependent	1 (12.5)	
Hypertension (%)		4 (50.0)	
Vascular disease (%)	No	5 (62.5)	
	Peripheral	0 (0.0)	
	Central	3 (32.5)	
Elevated creatinine level (%)		0 (0.0)	
Anticoagulants (%)		8 (100.0)	
Beta blocking agents (%)		1 (12.5)	
Renin–angiotensin–aldosterone system inhibitors (%)		2 (25.0)	
Calcium antagonist (%)		1 (12.5)	
Diuretics (%)		0 (0.0)	
Statins (%)		5 (62.5)	
Indication for cerebral bypass (%)	Moyamoya disease	5 (62.5)	
	Carotid occlusion	3 (37.5)	

*Intraoperative characteristics*		*Patients (N* = *8)*∗	*Cases (N* = *10)* †
Duration of surgery (min, median [IQR])		299 [260–357]	297 [260–342]
pH before start of study (mm Hg, median [IQR])		7.39 [7.38–7.40]	7.39 [7.38–7.40]
*P*aco_2_ before start of study (mm Hg, median [IQR])		5.2 [4.8–5.2]	5.2 [4.8–5.2]
ETCO_2_ before start of study (mm Hg, median [IQR])		4.8 [4.5–4.9]	4.8 [4.5–4.9]
Haemoglobin before start of study (g dl^−1^, median [IQR])		11.4 [9.8–12.1]	11.4 [10.0–11.9]
Haematocrit before start of study (%, median [IQR])		34 [29–35]	34 [30–35]
Baseline MAP before induction of anaesthesia (mm Hg, median [IQR])		98 [96–101]	98 [93–105]

**Fig 2:**
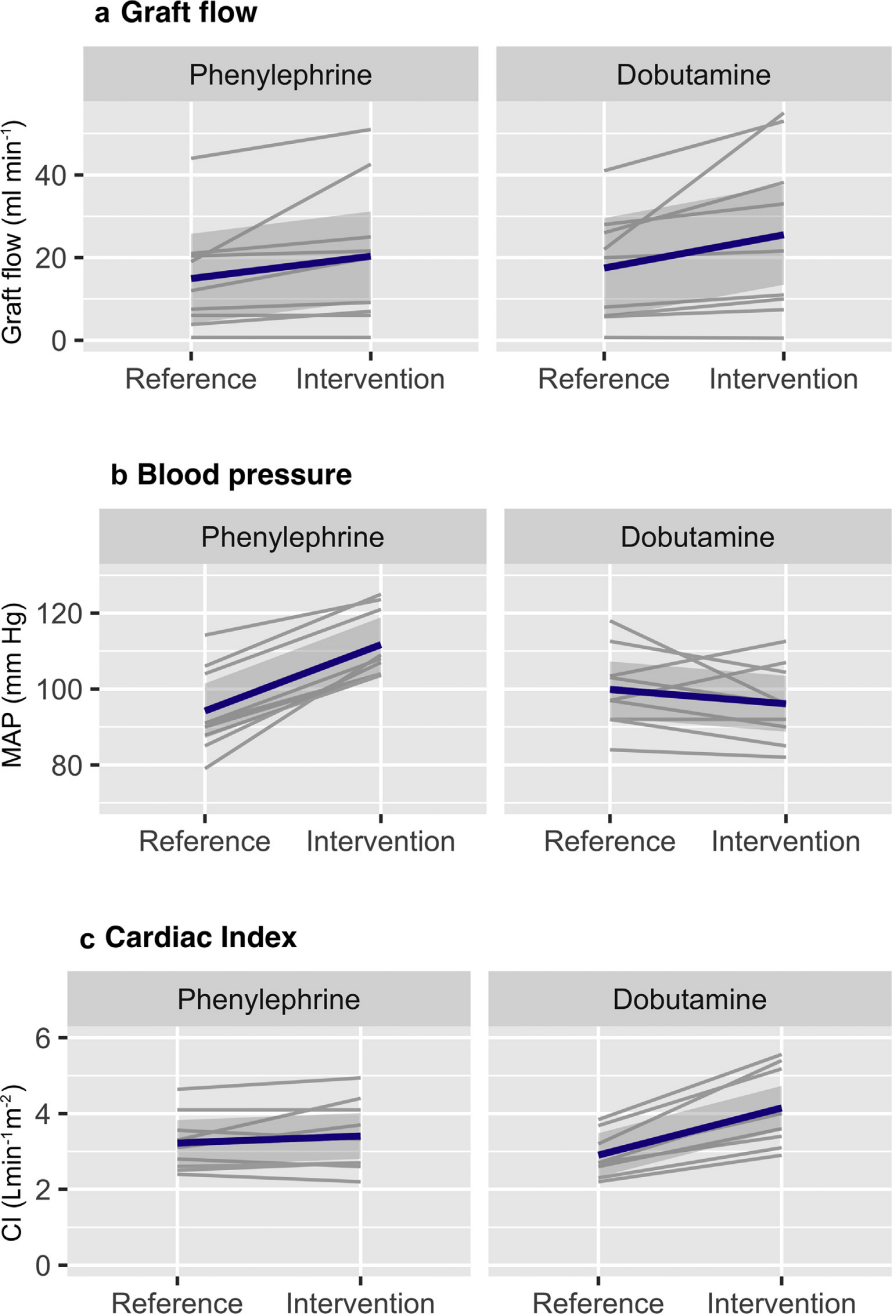
Spaghetti plot for change in graft flow, MAP, and cardiac index. The plots show the value for graft flow (a), mean arterial pressure (b) and cardiac index (c) for all patients. Two values were plotted per patient – that is the mean in the reference phase and the mean in the intervention phase. For visualisation purposes, spaghetti plots were made. The graphs on the left represent the results for the phenylephrine stage, whereas the graphs on the right represent the results obtained in the dobutamine stage. The blue line shows the mean change with standard deviation for all patients combined.

A median of 4.1 (IQR, 2.9–5.1) μg kg^−1^ min^−1^ dobutamine for a duration of 14 (IQR, 13–18) min and 0.31 (IQR, 0.26–0.41) μg kg^−1^ min^−1^ phenylephrine for 12 (10–14) min were administered. Graft flow increased with a median of 4.1 (IQR, 1.7–12.0) ml min^−1^ after administration of dobutamine and with 3.6 (IQR, 1.3–7.8) ml min^−1^ after administration of phenylephrine (Table 2). The pseudo-median difference in increase in graft flow of dobutamine *vs* phenylephrine was –0.6 ml min^−1^ (95% CI, –14.5 to 5.3). Cardiac index increased with a median of 1.1 (IQR, 0.8–1.5) L min^−1^ m^−2^ during dobutamine administration, whereas MAP decreased with a median of –7 (IQR, –7 to 0) mm Hg. MAP increased with a median of 16 (IQR, 14–19) mm Hg during phenylephrine administration, which was not accompanied by a change in cardiac index (median 0.1 [IQR, –0.2 to 0.3]) L min^−1^ m^−2^. In all patients, ETCO_2_ was kept constant (Supplementary Table S1). The change in graft flow, MAP, and cardiac index during dobutamine and phenylephrine administration is presented for all patients separately in Figure 2. After adjustment in the random effect multivariable linear regression model, type of treatment was not associated with graft flow (0.9 ml min^−1^; 95% CI, 0.0–20.1, *P*=0.944). There was no carry-over effect for dobutamine (0.0 ml min^−1^; 95% CI, 0.0–0.2, *P*=0.004).

**Table 2:** Change in haemodynamic parameters. Data from the dobutamine intervention in patient 6b were removed from the analyses, because arrhythmia occurred during dobutamine administration, making values obtained with the EV1000/FloTrac® system less reliable. ∗Statistically significant at a level of significance of *P*<0.05. ^†^Differences were estimated using a Wilcoxon signed rank test comparing the effects of dobutamine with phenylephrine and reporting a pseudomedian because differences between paired samples were not fully symmetrically distributed around the median. CI, confidence interval; IQR, inter-quartile range; MAP, mean arterial pressure; SBP, systolic blood pressure.

Haemodynamic parameters	Phenylephrine (median [IQR])			Dobutamine (median [IQR])			Between intervention difference (95% CI)^†^	*P*-value
	Reference	Intervention	Difference	Reference	Intervention	Difference		
*Cases (N* = *10)*								
Graft flow (ml min^−1^)	15.5 [6.4–20.9]	20.8 [7.5–32.5]	3.6 [1.3–7.8]	21.0 [6.4–27.5]	21.6 [10.0–38.2]	4.1 [1.7–12.0]	– 0.6 (–14.5 to 5.3)	0.441
MAP (mm Hg)	91 [88–101]	108 [104–118]	16 [14–19]	99 [93–103]	96 [90–104]	–7 [–7 to 0]	21 (12–31)	0.004∗
SBP (mm Hg)	139 [123–155]	169 [154–185]	32 [26–33]	146 [135–153]	151 [135–167]	5 [–1 to 20]	24 (7–35)	0.013∗
Cardiac index (L min^−1^ m^−2^)	3.0 [2.6–3.5]	3.0 [2.6–4.0]	0.1 [–0.2 to 0.3]	2.7 [2.4–3.1]	4.0 [3.4–5.2]	1.1 [0.8–1.5]	–1.0 (–1.4 to –0.7)	0.009∗
Heart rate (beats min^−1^)	53 [50–59]	51 [47–59]	–1 [–2 to –1]	53 [50–57]	62 [57–64]	5 [2–6]	–7 (–12 to –4)	0.004∗
Stroke volume (ml)	57 [49–63]	60 [55–68]	4 [–0.5 to 7]	54 [44–63]	69 [62–80]	13 [11–22]	–10 (–19 to –4)	0.004∗

In one patient, distinctive results were found. This patient was included twice as she presented for a second, contralateral bypass. During the second procedure we found a very low graft flow, with hardly any effect on graft flow during both an increase in MAP and cardiac index (Supplementary Table S1). A CT angiography revealed a large collateral system surrounding the circle of Willis, shunting blood away from the bypass. To determine whether hypovolaemia might also have affected the results found in this patient, we retrospectively collected data on pulse pressure variation from our anaesthesia record-keeping system and found no clinically relevant change (Supplementary Table S2).

## Discussion

We administered dobutamine and phenylephrine to increase cardiac output and blood pressure, respectively, during cerebral bypass surgery and observed similar increases in graft flow. This finding was consistent in most patients and except for a short episode of atrial arrhythmia in one patient, no adverse effects were observed.

### Blood pressure, cardiac output, and cerebral perfusion

When assessing the effect of blood pressure and cardiac output on cerebral perfusion, studies have differentiated between awake and anaesthetised patients and between healthy patients and patients with a disturbed cerebral autoregulation. A study in healthy patients under general anaesthesia found that an increase in MAP with phenylephrine caused a decrease in cerebral oxygenation as measured with near-infrared spectroscopy (NIRS), whereas the cardiac output did not change.^9^ A similar increase in MAP after ephedrine administration increased cardiac output and preserved cerebral oxygenation.^9^ Although this finding suggests that cardiac output may be an important variable to maintain cerebral oxygenation, interpretation is complicated because NIRS was used to assess cerebral oxygenation.^8^ The accuracy of NIRS is known to suffer from extracranial contamination and is influenced by vasopressors causing vasoconstriction of the scalp.^18^ Studies using healthy, awake patients found that 1% change in cardiac output corresponded to a 0.35% change in cerebral blood flow velocity, as measured with transcranial Doppler.^10^, ^11^, ^12^^,^^19^

However, results from studies in awake and healthy subjects may not always apply to the diseased population under general anaesthesia. Anaesthesia can potentially affect cerebral perfusion via a variety of pathways, including suppression of the sympathetic nervous system.^12^ However, anaesthesia does not seem to significantly affect cerebral autoregulation itself in patients with intact or disrupted cerebral autoregulation.^20^, ^21^, ^22^ It appears that the cerebral vasculature also does not respond differently on vasoactive agents after initiation of general anaesthesia.^22^^,^^23^

Healthy subjects are likely to have good cerebral autoregulation, whereas the effects of adrenergic agents may be stronger in patients with disrupted cerebral autoregulation.^8^ This is supported by a study in healthy patients where maintenance of MAP with phenylephrine after initiation of general anaesthesia decreased cerebral oxygenation^9^, whereas a comparable study in patients with disrupted autoregulation found maintained cerebral oxygenation.^24^

The differential effects of blood pressure and cardiac output on cerebral blood flow in the neurosurgical population is insufficiently studied. Interestingly, cerebral dysregulation is a phenomenon also seen cardiac surgery.^8^^,^^25^ A randomised trial studying the effect of a low target MAP (40–50 mm Hg) *vs* a high target MAP (70–80 mm Hg) during cardiopulmonary bypass with a fixed bypass flow found no difference in new ischaemic cerebral lesions on MRI.^26^ Another study in cardiac surgery patients found that cerebral oxygenation was lower with lower pump flow, regardless of arterial blood pressure.^27^ Like the studies in cardiac patients, the present study found that an increase in cardiac output can increase cerebral blood flow, even when MAP remains unchanged.

### Clinical implications

Although cerebral bypass surgery aims to prevent future ischaemic strokes, patients are at risk for perioperative cerebral ischaemia.^1^ Currently, vasopressors are used to maintain blood pressure levels.^3^^,^^28^ However, in patients with a disrupted autoregulation the cerebral perfusion may not solely depend on blood pressure, as demonstrated in studies conducted in the cardiac surgery population^12^^,^^26^^,^^27^ and supported by findings in the present study. It should be noted that the effects of dobutamine and phenylephrine on cerebral blood flow – via cardiac output and blood pressure – cannot be interpreted separately from the direct effect of these agents on cerebral vessels and the close relation between cardiac output and blood pressure.

Both α-adrenergic and β-adrenergic receptors are suggested to play a role in cerebral autoregulation.^8^^,^^29^^,^^30^ Vasopressors used to increase blood pressure, such as the α_1_-receptor agonist phenylephrine, might actually cause cerebral vasoconstriction.^31^ We propose that the increase in cardiac output by dobutamine caused the increase in graft flow found in this study, while only having a minimal α-adrenergic effect at best, thus preventing cerebral vasoconstriction. However, dobutamine can also decrease blood pressure to a varying extent, possibly explaining why not all patients benefitted from dobutamine administration. In addition, by increasing preload, administration of phenylephrine can also increase cardiac output when anaesthesia-induced hypotension is the result of hypovolaemia.^32^ Interestingly, the present study found that phenylephrine did not change cardiac output. In addition, others found a decrease in cardiac output after phenylephrine administration.^33^ These differences might be explained by timing of phenylephrine administration (i.e. anaesthesia-induced hypotension might be maximal immediately after initiation of anaesthesia) and the method of cardiac output measurement.^32^, ^33^, ^34^, ^35^ Our study used a fourth-generation FloTrac® algorithm for arterial pressure waveform analysis, with good performance when reporting changes in cardiac output after phenylephrine administration.^33^, ^34^, ^35^

We cannot definitively conclude that dobutamine administration benefits all patients presenting for cerebral bypass surgery. Still, this study does show that dobutamine can increase cerebral perfusion and should be considered when targeted graft flows are not reached or only at the cost of (severe) systemic hypertension, when using phenylephrine. Inotropes such as dobutamine can cause arrhythmia and increased myocardial oxygen demand and should be used with caution in patients with cardiac comorbidities. Although not tested in this study, norepinephrine, a combined α_1_-, α_2_-and β_1_-receptor agonist, primarily causes vasoconstriction and an increase in blood pressure, but can also (slightly) increase cardiac output and may be a good alternative to phenylephrine.^36^ Future studies should consider testing the effect of norepinephrine on cerebral blood flow and further explore the effect of an increase in cardiac output in all patients at risk of perioperative cerebral ischaemia.

### Strengths and limitations

This study has several strengths. First, cerebral bypass procedures provided us the opportunity to measure cerebral perfusion invasively with an ultrasonographic flow meter in close proximity to the middle cerebral artery, providing reliable measurements.^37^ In contrast, several other existing techniques, such as NIRS, each have their limitations, ranging from invasiveness to contamination by the extracranial circulation.^8^ Doppler sonography assesses velocity (cm s^−1^) rather than volume per time unit (ml min^−1^), and is no longer reliable when the diameter of the vessel changes.^8^ Second, as *P*aco_2_ levels are known to influence cerebral blood flow, it is important to keep these constant, which was confirmed by stable ETCO_2_ levels throughout the conduct of the study. Third, owing to the crossover design of this study we were able to eliminate substantial between-patient variability. Finally, a wash-out period was used between both interventions and there was no carry-over effect.

Nevertheless, this study has several obvious limitations. First, most patients suffered from moyamoya disease, which limits generalisability. However, the results from this study might also be applicable to a broader population at risk for perioperative cerebral ischaemia. Like in moyamoya disease, cardiac surgery patients, post-ischaemic stroke patients and patients after subarachnoid haemorrhages or traumatic brain injury all suffer from a disrupted autoregulation.^8^^,^^25^^,^^38^^,^^39^ However, to confirm or refute such effects in other patient populations, targeted cerebral blood flow studies are needed, administering vasoactive drugs in a similar crossover design as used in the present study. Second, an increase in cardiac index and MAP of 10–20% was intended. The increase in cardiac index was much higher than intended. However, this further strengthens our observation that dobutamine was not superior to phenylephrine in improving cerebral perfusion. Third, we used an uncalibrated sensor to continuously measure cardiac index. The accuracy of the EV1000/FloTrac® system has been shown to be sufficient in the absence of large changes in vascular tone and can be used to follow trends in cardiac index over time.^14^^,^^35^

Both administration of dobutamine, by increasing cardiac output while decreasing MAP, and phenylephrine, by increasing MAP while maintaining cardiac output, increased graft flow in patients undergoing cerebral bypass surgery.

## Author contributions

Study design: AA, JARvW, TPCD, EECdW, AvdZ, CJK, WAvK

Patient recruitment: AA

Data collection: AA

Data analysis: AA, JARvW

Data interpretation: AA, JARvW, TPCD, GJER, AvdZ, CJK, WAvK

Drafting of the manuscript: AA, JARvW, WAvK

Critical revision of the manuscript: JARvW, TPCD, EECdW, GJER, AvdZ, CJK, WAvK

Final approval of the manuscript: all authors.

## Declarations of interest

The authors declare that they have no conflicts of interest.

## Funding

Departmental sources.
