# Supplementary material for: Effects of dobutamine and phenylephrine on cerebral perfusion in patients undergoing cerebral bypass surgery: a randomised crossover trial
Source: Br J Anaesth. 2020 Jul 24;125(4):539–47. doi: 10.1016/j.bja.2020.05.040 (PMC7565906; doi:10.1016/j.bja.2020.05.040)
Supplement: Multimedia component 1 [file mmc1.doc]

**CONSORT 2010 Flow Diagram**

**Allocation**

**Analysis**

**Follow-Up**

**Enrollment**

Assessed for eligibility (n=17)

Excluded (n=7)

♦  Not meeting inclusion criteria (n=0)

♦  Declined to participate (n=6)

♦  Other reasons (n=1)

Analysed (n=4)
♦ Excluded from analysis (give reasons) (n=0)

*One patient developed arrhythmia after dobutamine administration. Only data obtained during the dobutamine intervention were excluded from the analyses.*

Lost to follow-up (give reasons) (n=0)

Discontinued intervention (give reasons) (n=0)

Allocated to intervention (n=4)

♦ Received allocated intervention (n=4)

♦ Did not receive allocated intervention (give reasons) (n=0)

Lost to follow-up (give reasons) (n=0)

Discontinued intervention (give reasons) (n=0)

Allocated to intervention (n=6)

♦ Received allocated intervention (n=6)

♦ Did not receive allocated intervention (give reasons) (n=0)

Analysed (n=6)
♦ Excluded from analysis (give reasons) (n=0)

Randomized (n=10)
